# Supplementary material for: A live cell reporter of exosome secretion and uptake reveals pathfinding behavior of migrating cells
Source: Nat Commun. 2020 Apr 29;11:2092. doi: 10.1038/s41467-020-15747-2 (PMC7190671; doi:10.1038/s41467-020-15747-2)
Supplement: Supplementary file 1 — Supplementary Information [file 41467_2020_15747_MOESM1_ESM.pdf]

Supplementary Information

A live cell reporter of exosome secretion and uptake reveals pathfinding behavior of migrating cells

Sung et al.

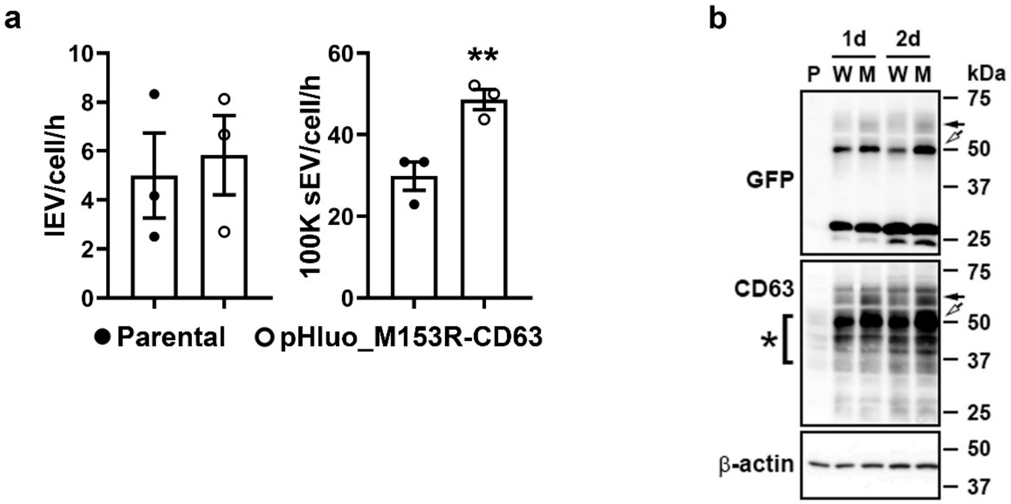

Supplementary Figure 1. Overexpression of pHluorin_M153R-CD63 increases secretion rate of exosome-like small EVs. (**a**) EV secretion rates measured by NTA. lEV, large EV. sEV, small EV. Mean +/- standard error from three independent experiments. ***P*=0.00409 compared with parental cell line by two-sided paired Student's *t*-test. (**b**) Western blots for transiently transfected HT1080 cells. P, parental cell lysate. W, pcDNA-pHluorin-CD63-transfected cell lysate. M, pcDNA-pHluorin_M153R-CD63-transfected cell lysate. 1d, 1 day after transfection. 2d, 2 days after transfection. Black arrows indicate full length pHluorin_M153R-tagged CD63, which is shifted due to the GFP moiety of 27 kDa, while white arrows indicate potential cleaved form of CD63 tagged with pHluorin_M153R. Asterisk indicates cellular CD63, which has a broad range due to glycosylation. Source data are provided as a Source Data file.

a

MDA-MB-231

b

SCC-61

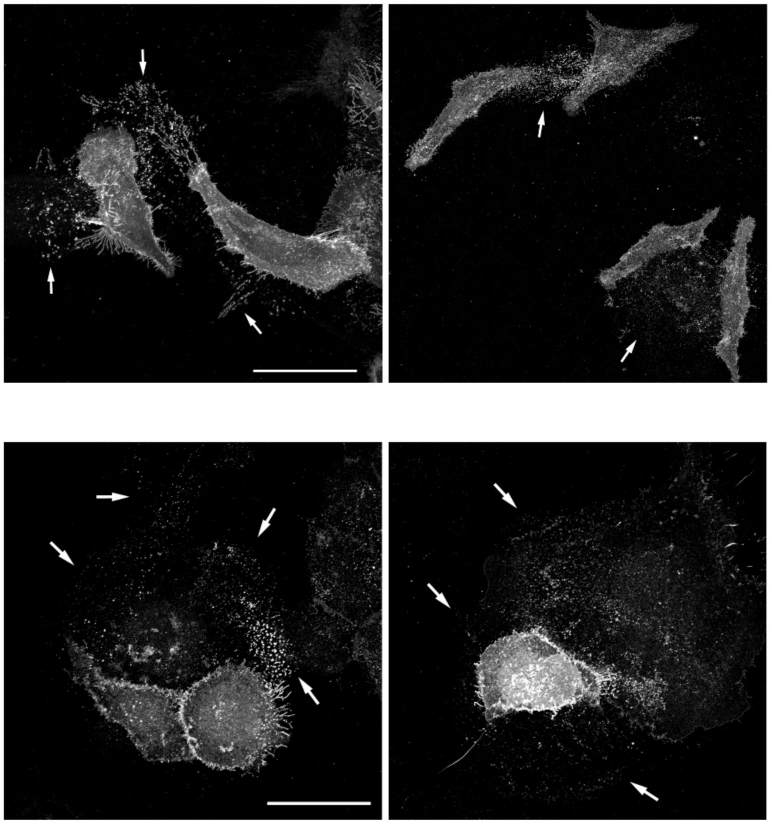

Supplementary Figure 2. pHluorin_M153R-CD63-positive trails are deposited from diverse cancer cell types. (**a**) Representative fluorescence images of live MDA-MB-231 human breast cancer cells stably expressing pHluo_M153R-CD63 from 4 independent experiments. Scale bar, 50 µm. (**b**) Representative fluorescence images of live HNSCC-61 human head and neck cancer cells stably expressing pHluo_M153R-CD63 from 4 independent experiments. Scale bar, 50 µm.

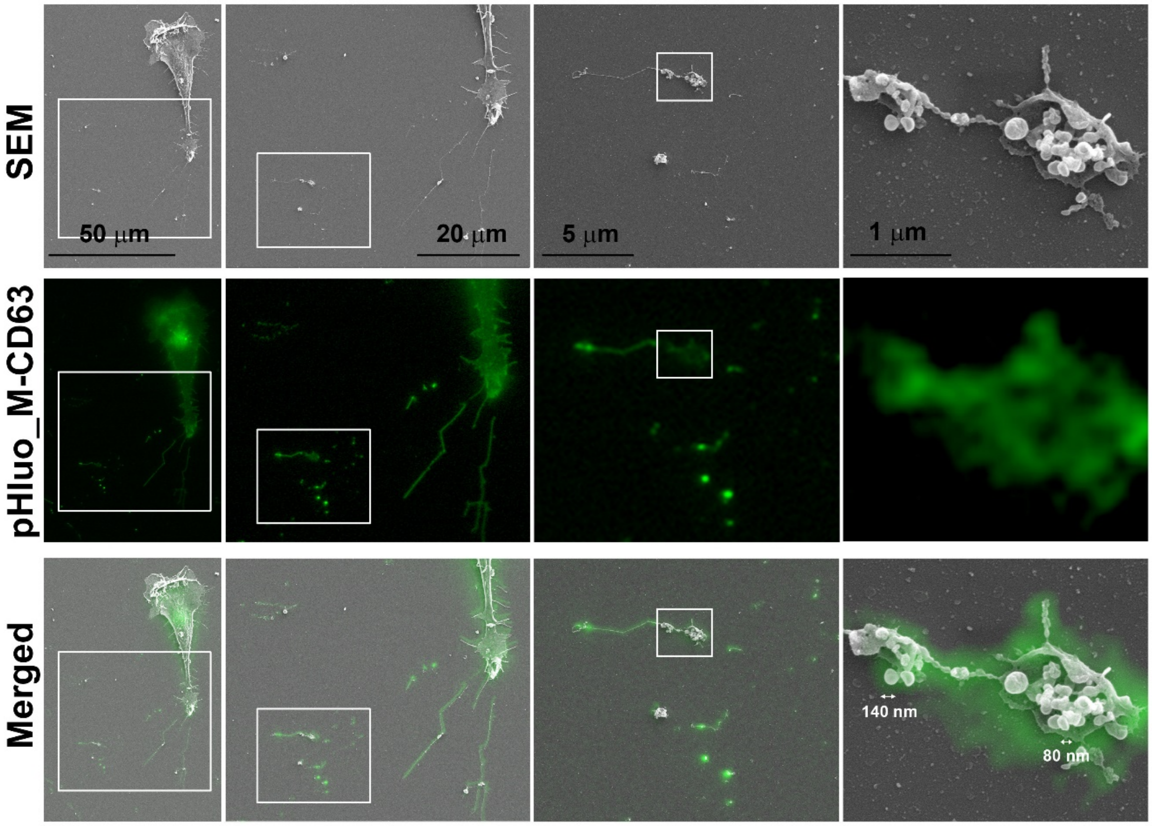

Supplementary Figure 3. pHluorin_M153R-CD63-positive trails mark exosome deposits. Correlative light-electron microscopy of pHluo_M153R-CD63 (green)-stably expressing HT1080. Zoom-in images from the left panel are shown to the right panel. 3 pouches were found from 4 independent experiments as described in the Fig. 3 legend.

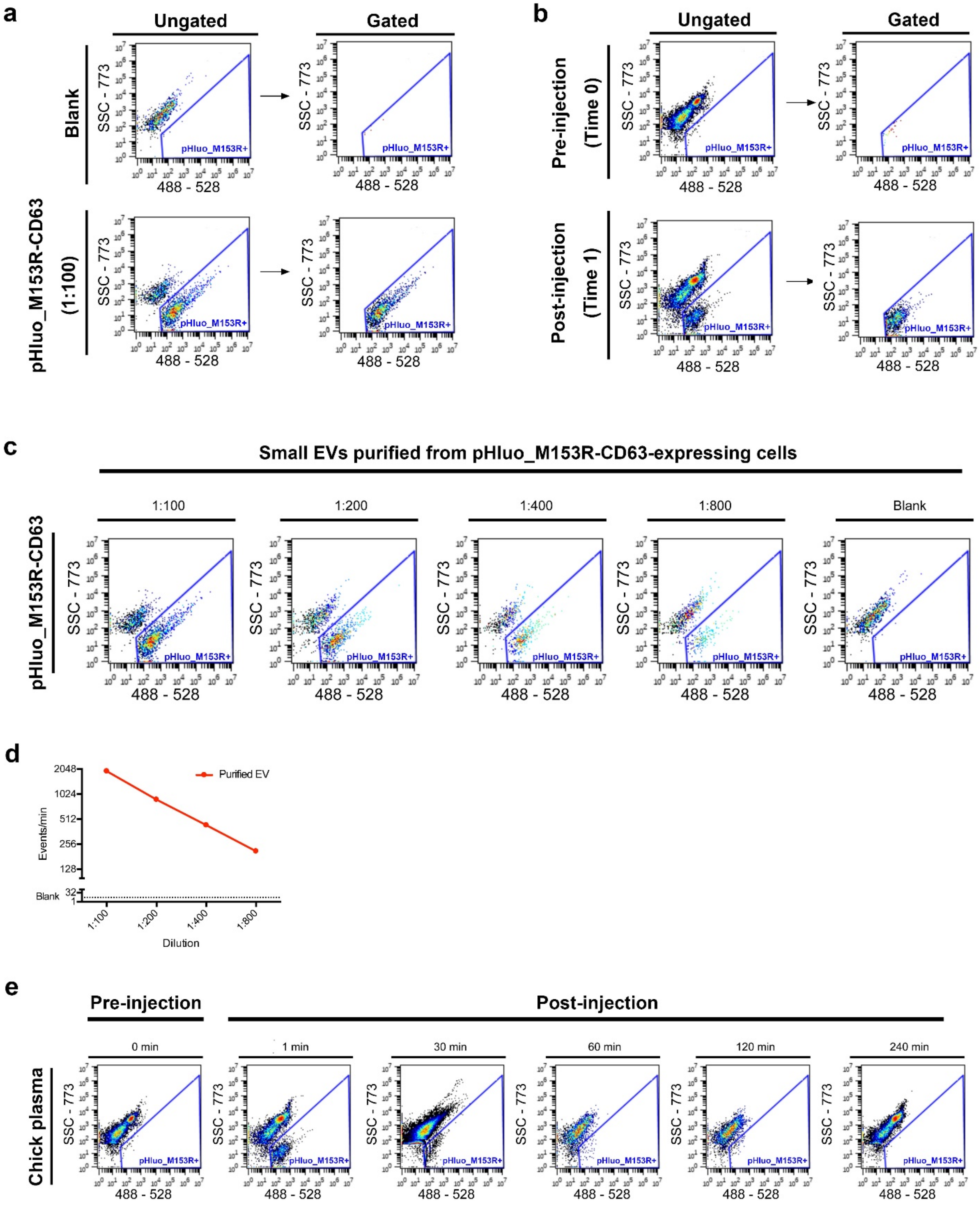

Supplementary Figure 4. Flow cytometry gating strategy for pHluo_M153R-CD63 EV detection in plasma. (**a**) Gating of pHluo_M153R-CD63 positive events (pHluo_M153R+) using blank flow buffer (top) and the 1:100 dilution of 100K-pHluo_M153R-CD63 EVs in flow buffer (bottom). (**b**) Gating of plasma background using pre-injection (Time 0 min, top) and post-injection (Time 1 min, bottom) chick

plasma used for half-life determination presented in Fig. 4g. The same gate was set to include pHluo_M153R+ events from (**a**) and exclude plasma background. (**c**) Two-fold dilution series of 100K-pHluo_M153R-CD63 EVs in flow buffer. (**d**) Quantification of gated events from (**c**) at the corresponding dilutions. (**e**) Detection of fluorescent 100K-pHluo_M153R-CD63 EVs over time. Pre-injection at time 0 min denotes blank plasma collected from the same chick, while post-injection plasma indicates plasma collected after intravenous injection of 100K-pHluo_M153R-CD63 EVs at 1 min, 30 min, 60 min, 120 min, and 240 min.

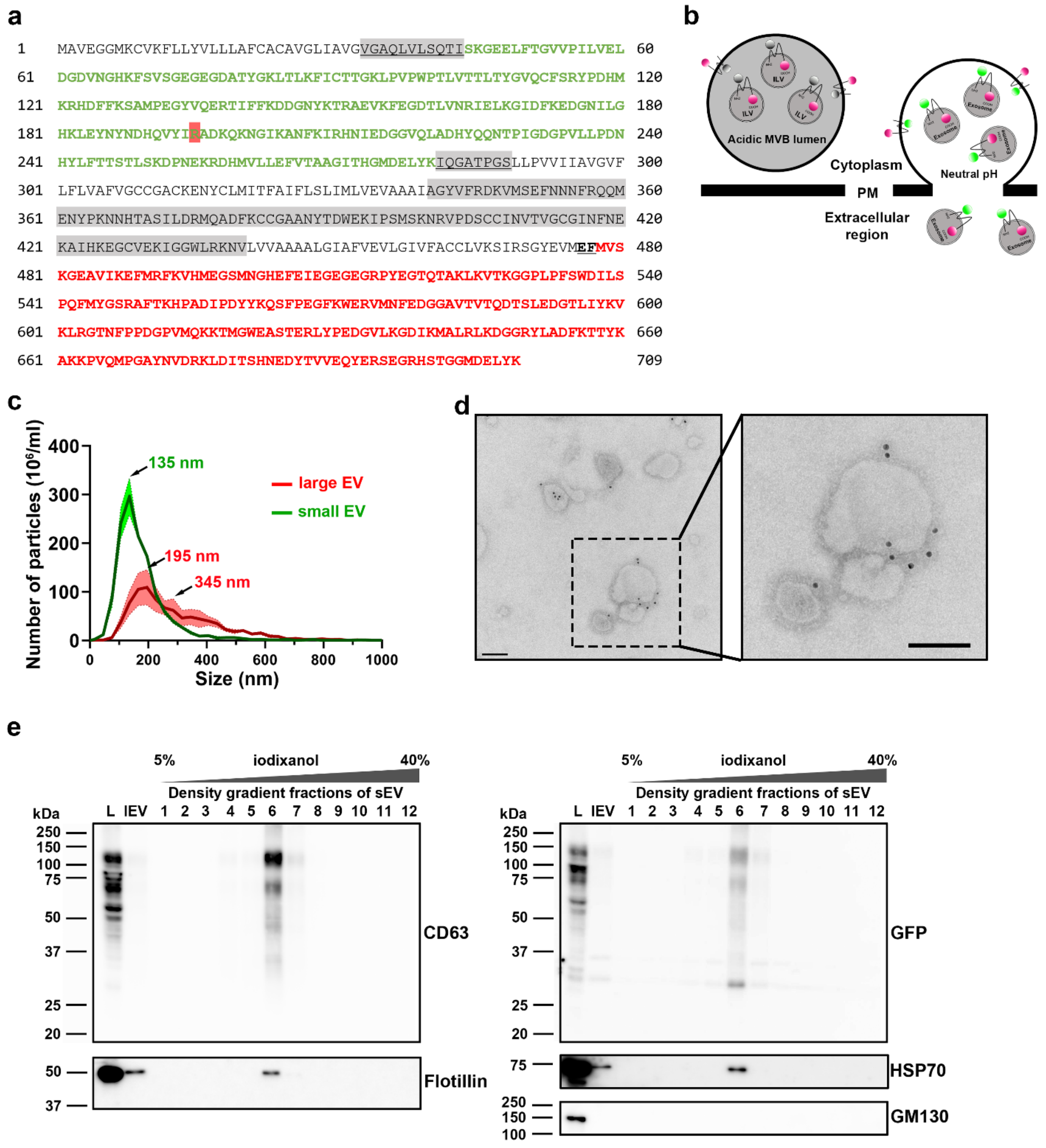

Supplementary Figure 5. pHluorin_M153R-CD63-mScarlet is a dual tagged multivesicular body and exosome reporter. (**a**) Amino acid sequence of pHluorin_M153R-CD63-mScarlet. pHluorin sequence is in green color. Highlighted regions in grey represent small (underlined) and large extracellular loops. M153R mutation is highlighted in red. Two extra amino acids between C-terminus of CD63 and N-terminus of mScarlet are underlined in bold. mScarlet sequence is marked in red. (**b**) Diagram of pHluorin_M153R-CD63-mScarlet construct. Note that unlike the pHluorin_M153R tag, mScarlet tag has bright fluorescence in the MVB as well as in small EVs regardless of pH. ILV, intraluminal vesicle. PM,

plasma membrane. (**c**) Averaged trace from nanoparticle tracking analyses of large EVs (lEVs) and small EVs (sEVs) secreted from pHluo_M153R-CD63-mScarlet expressing HT1080. Shaded area represents s.e.m. from three independent experiments. (**d**) Representative immunogold negative stain electron micrograph of small EVs purified by iodixanol density gradient from pHluorin_M153R-CD63-mScarlet-expressing HT1080 cells from 2 independent experiments. Scale bar, 100 nm. (**e**) Western blot analysis of cells and EVs with anti-CD63, anti-GFP, EV markers (Flotillin, HSP70) and Golgi marker (GM130). L, total cell lysate. lEV, large EV. sEV, small EV. Source data are provided as a Source Data file.
