# Supplementary material for: A live cell reporter of exosome secretion and uptake reveals pathfinding behavior of migrating cells
Source: Nat Commun. 2020 Apr 29;11:2092. doi: 10.1038/s41467-020-15747-2 (PMC7190671; doi:10.1038/s41467-020-15747-2)
Supplement: Supplementary file 3 — Description of Additional Supplementary Files [file 41467_2020_15747_MOESM3_ESM.pdf]

Description of Additional Supplementary Files

File Name: Supplementary Movie 1
Description: Migrating HT1080 cell stably expressing pHluo_M153R-CD63 and mCherry-CaaX (upper panel) and transiently expressing pHluo-CD63 (lower panel). Time-lapse images were taken on a fibronectin-coated MatTek dish (1 µg ml^{-1}) every 30 sec. Note the extracellular puncta left behind the migrating cell and the dimmer fluorescence from pHluo-CD63 compared with pHluo_M153R-CD63. Scale bars, 50 µm.

File Name: Supplementary Movie 2
Description: Migrating HT1080 cells in a 2D environment exhibit pathfinding behavior over pHluo_M153R-CD63 deposits. Time-lapse images were taken on a fibronectin-coated MatTek dish (1 µg ml^{-1}) every 5 min. Scale bar, 50 µm.

File Name: Supplementary Movie 3
Description: Migrating HT1080 cells in a 3D environment exhibit pathfinding behavior over pHluo_M153R-CD63 deposits. Time-lapse images were taken in collagen gels (1.5 mg ml^{-1}) every 10 min. Scale bar, 50 µm.

File Name: Supplementary Movie 4
Description: 3-dimensional reconstruction of pHluo_M153R-CD63 deposition in a mouse mammary tissue shown in Fig 4e. Arrows indicate pHluo_M153R-CD63 deposits.

File Name: Supplementary Movie 5
Description: Live confocal imaging of pHluo_M153R-CD63 on a polymeric nanopatterned dish. Time-lapse images were taken every minute. Note that pHluo_M153R-CD63 deposits at the retraction fibers stayed stationary as the cells moved over them. Scale bars, 10 µm.

File Name: Supplementary Movie 6
Description: Live confocal imaging of pHluo_M153R-CD63-mScarlet reveals that MVBs are trafficked toward and exosomes are secreted at the leading edge of migrating cells. Time-lapse images were taken every 10 sec. The white rectangle across the leading edge indicates the region for the kymograph shown in Fig. 7a. Scale bar, 50 µm.

File Name: Supplementary Movie 7
Description: Endocytosis and acidification of extracellular exosome deposits. Live confocal imaging of pHluo_M153R-CD63-mScarlet with frames taken every minute. Note that the HT1080 cell migrates toward the exosome deposits and contacts them using filopodia. Contacted exosome deposits (white arrows) are endocytosed and acidified in endosomal compartments (magenta arrows). Scale bar, 25 µm.
