# Supplementary material for: A live cell reporter of exosome secretion and uptake reveals pathfinding behavior of migrating cells
Source: Nat Commun. 2020 Apr 29;11:2092. doi: 10.1038/s41467-020-15747-2 (PMC7190671; doi:10.1038/s41467-020-15747-2)
Supplement: Supplementary file 11 — Reporting Summary [file 41467_2020_15747_MOESM11_ESM.pdf]

nature research

Corresponding author(s): Alissa Weaver

Last updated by author(s): Mar 4, 2020

Reporting Summary

Nature Research wishes to improve the reproducibility of the work that we publish. This form provides structure for consistency and transparency in reporting. For further information on Nature Research policies, see Authors & Referees and the Editorial Policy Checklist.

Statistics

For all statistical analyses, confirm that the following items are present in the figure legend, table legend, main text, or Methods section.

n/a	Confirmed	
☐	☒	The exact sample size (*n*) for each experimental group/condition, given as a discrete number and unit of measurement
☐	☒	A statement on whether measurements were taken from distinct samples or whether the same sample was measured repeatedly
☐	☒	The statistical test(s) used AND whether they are one- or two-sided<br>*Only common tests should be described solely by name; describe more complex techniques in the Methods section.*
☒	☐	A description of all covariates tested
☐	☒	A description of any assumptions or corrections, such as tests of normality and adjustment for multiple comparisons
☐	☒	A full description of the statistical parameters including central tendency (e.g. means) or other basic estimates (e.g. regression coefficient) AND variation (e.g. standard deviation) or associated estimates of uncertainty (e.g. confidence intervals)
☐	☒	For null hypothesis testing, the test statistic (e.g. *F*, *t*, *r*) with confidence intervals, effect sizes, degrees of freedom and *P* value noted<br>*Give P values as exact values whenever suitable.*
☒	☐	For Bayesian analysis, information on the choice of priors and Markov chain Monte Carlo settings
☒	☐	For hierarchical and complex designs, identification of the appropriate level for tests and full reporting of outcomes
☒	☐	Estimates of effect sizes (e.g. Cohen's *d*, Pearson's *r*), indicating how they were calculated

Our web collection on statistics for biologists contains articles on many of the points above.

Software and code

Policy information about availability of computer code

Data collection	NIS-Elements (5.20.02), MetaMorph (7.7.6.0), ZetaView (8.04.02), ZEN (8.0), PrairieView (5.4), Volocity (5.5), CellStream Acquisition (1.2.87)
Data analysis	NIS-Elements (5.20.02), Fiji (1.52p), MetaMorph (7.7.6.0), Imaris (9.2.1), Microsoft Excel (Office 2019), GraphPad Prism 8 (8.2.1), Cytobank (7.2)

For manuscripts utilizing custom algorithms or software that are central to the research but not yet described in published literature, software must be made available to editors/reviewers. We strongly encourage code deposition in a community repository (e.g. GitHub). See the Nature Research guidelines for submitting code & software for further information.

Data

Policy information about availability of data

All manuscripts must include a data availability statement. This statement should provide the following information, where applicable:

- Accession codes, unique identifiers, or web links for publicly available datasets
- A list of figures that have associated raw data
- A description of any restrictions on data availability

The data supporting the findings of this study are available from the corresponding author upon reasonable request.

Field-specific reporting

Please select the one below that is the best fit for your research. If you are not sure, read the appropriate sections before making your selection.

☒ Life sciences ☐ Behavioural & social sciences ☐ Ecological, evolutionary & environmental sciences

Life sciences study design

All studies must disclose on these points even when the disclosure is negative.

Sample size	Most of our data is imaging data. We perform at least 3 independent experiments and capture multiple images or movies on multiple cells. We specify the number of cells and independent experiments in the figure legends. We have experience in approximately how many cells are needed to be quantitated for immunofluorescence staining and motility experiments (Sung et al. Nature Communcations 6, Article number: 7164 (2015)). We also can add independent replicates if necessary.<br><br>For the intravital imaging from mice, the goal with pHluorin_M153R-CD63 expressing MDA-MB-231 cells was to visually identify secreted exosomes during cell migration in vivo rather than to quantify any specific aspect of this process. Thus, the experimental cohort consists of a total of 8 mice (tumors).<br><br>For the avian embryo experiments, initial assay conditions were established by diluting purified pHluo_M153R-CD63-labeled EVs into chick plasma. Using those conditions, we analyzed plasma obtained from chick embryos before and after injection of purified pHluo_M153R-CD63-containing EVs. Since pHluorin is not naturally present in chicks, the sample size was defined by the variation across injected chicks. Based on successful prior analysis of chick embryos (Zijlstra et. al. Cancer Cell. 2008 Mar;13(3):221-34, Sung et. al., Nature Communication. 2015 May 13;6:7164, Hebron et. al. Scientific Report. 2018 Feb 16;8(1):3208) we performed 3 separate experiments, each containing 3 animals.
Data exclusions	We have experience which data should be excluded (Sung et al. Nature Communcations 6, Article number: 7164 (2015)) and only exclude analyses if there is an obvious reason for poor data, such as dividing, dead or sick-looking cells. For example, dividing cells were excluded for cell migration analysis since the cell division affects single cell motility.<br><br>For the avian experiments we eliminated animals for which fewer than 3 consecutive bleeds could be analyzed.
Replication	All statistically analyzed experiments were performed at least 3 times independently. All attempts at replication were successful.
Randomization	Cellular experiments including fluorescent microscopy, electron microscopy, cell motility analyses were analyzed based on randomly selected fields.
Blinding	Investigators were not blinded in the data analyses since the same personnel did the associated experiments and blinding was not necessary to this study.

Reporting for specific materials, systems and methods

We require information from authors about some types of materials, experimental systems and methods used in many studies. Here, indicate whether each material, system or method listed is relevant to your study. If you are not sure if a list item applies to your research, read the appropriate section before selecting a response.

Materials & experimental systems

n/a	Involved in the study	
☐	☒	Antibodies
☐	☒	Eukaryotic cell lines
☒	☐	Palaeontology
☐	☒	Animals and other organisms
☒	☐	Human research participants
☒	☐	Clinical data

Methods

n/a	Involved in the study	
☒	☐	ChIP-seq
☐	☒	Flow cytometry
☒	☐	MRI-based neuroimaging

Antibodies

Antibodies used	anti-CD63 (ab68418, abcam, 1:500 for WB and ab8219, abcam, 1:100 for IF), anti-GFP (A11122, Invitrogen, 1:5,000 for WB and 1:500 for immunogold TEM), anti-TSG101 (ab30871, abcam,1:1,000 for WB and 1:100 for IF), anti-flotillin (610820, BD Biosciences, 1:1,000 for WB), anti-HSP70 (sc-373867, Santa Cruz Biotechnology, 1:1,000 for WB), anti-GM130 (610822, BD Biosciences, 1:250 for WB), anti-Rab27a (69295, Cell Signaling, 1:1,000 for WB), anti-Alix (2171, Cell Signaling, 1:200 for IF), anti-LAMP1 (555798, BD Biosciences, 1:500 for IF), anti-Rab7 (9367, Cell Signaling, 1:200 for IF), anti-β-actin (Ac-74, Sigma, 1:5,000 for WB), HRP-conjugated mouse IgG (W4021, Promega, 1:10,000 for WB), HRP-conjugated rabbit IgG (W4011, Promega, 1:10,000 for WB), AlexaFluor 546-conjugated donkey anti-mouse IgG (A10036, Invitrogen 1:1,000 for IF) or goat anti-rabbit IgG (A11010, Invtrogen 1:1,000 for IF), AlexaFluor 647-conjugated donkey anti-mouse IgG (A31571, Invitrogen, 1:1,000 for IF) or goat anti-rabbit IgG (A21244, Invitrogen, 1:1,000 for IF), and colloidal gold (10 nm)-conjugated donkey anti-rabbit IgG from Electron Microscopy Sciences (25704, 1:40 for immunogold TEM).
Validation	CD63 Ab: Knockout tested by the manufacturer and cited in 24 publications according the manufacturer. ab68418 is suitable for WB. ab8219 is suitable for Flow Cytometry (Flow Cyt), Immunohistochemistry (Formalin/PFA-fixed parafin-embedded section, IHC-P), and Immunocytochemistry/Immunofluorescence (ICC/IF). Both antibodies react with human based on the manufacturer's website.

GFP Ab: Tested with GFP-overexpressing cell lysates by the manufacturer and cited in 1592 publications according the manufacturer. Suitable for ICC (Paraffin and Frozen), IF, IHC, WB, Immunoprecipitation (IP), ChIP Assay (ChIP), Flow Cyt, Gel Shift, In Situ Hybridization (ISH), ELISA, and Neutralization based on the manufacturer's website.
TSG101 Ab: Validated by the manufacturer and cited in 47 publications according to the manufacturer. Suitable for WB, ICC/IF, and IHC-P. Reactive with mouse, rat, and human based on the manufacturer's website.
Flotillin Ab: Validated by the manufacturer and and cited in 158 publications according BenchSci, an AI-assisted antibody selection platform. Suitable for WB and IF. Reactive with rat, human, mouse, chicken based on the manufacturer's website.
HSP70 Ab: Validated by the manufacturer and cited in 7 publications according to the manufacturer. Suitable for WB, IP, IF, IHC-P and ELISA. Reactive with mouse, rat, human, canine, bovine, and porcine based on the manufacturer's website.
GM130 Ab: Validated by the manufacturer and and cited in 158 publications according BenchSci, an AI-assisted antibody selection platform. Suitable for WB, IF, and IP. Reactive with rat, human, mouse, and dog based on the manufacturer's website.
Rab27a Ab: Validated by the manufacturer and cited in 3 publications according to the manufacturer.
Alix Ab: Validated by the manufacturer and cited in 60 publications according to the manufacturer. Suitable for WB and IP. Reactive with human, mouse, rat, and monkey based on the manufacturer's website. Usage for IF was published (Miao et al. Cell 2015; 161(6): 1306-1319).
LAMP1 Ab: Validated by the manufacturer and and cited in 33 publications according BenchSci, an AI-assisted antibody selection platform. Suitable for IF and Flow Cyt. Reactive with human based on the manufacturer's website.
Rab7 Ab: Validated by the manufacturer and cited in 166 publications according to the manufacturer. Suitable for WB, IP, and IF. Reactive with human, mouse, rat, and monkey based on the manufacturer's website.
β-actin Ab: Validated by the manufacturer and cited in 1219 publications according to the manufacturer. Suitable for ELISA, IF, IHC, and WB. Reactive with Drosophila, Hirudo medicinalis, carp, rabbit, wide range, pig, cat, human, rat, chicken, guinea pig, sheep, mouse, bovine, and canine based on the manufacturer's website.

Eukaryotic cell lines

Policy information about cell lines

Cell line source(s): HT1080 and MDA-MB-231 were purchased from ATCC. HNSCC61 was a gift from Wendell Yarbrough (University of North Carolina at Chapel Hill School of Medicine)

Authentication: None of the cell lines were authenticated.

Mycoplasma contamination: All cell lines were regularly mycoplasma-tested and negative.

Commonly misidentified lines (See ICLAC register): None

Animals and other organisms

Policy information about studies involving animals; ARRIVE guidelines recommended for reporting animal research

Laboratory animals: 8 week old female NOD/SCID mice and 10-15 day-old chick embryos

Wild animals: This study did not involve wild animals.

Field-collected samples: This study did not involve samples collected from the field.

Ethics oversight: All mouse imaging and surgical protocols were approved by the University of Wisconsin Institutional Animal Care and Use Committee.
Prior to day 17 the chick embryo is not considered a "vertebrate animal" and is therefore managed as BSL-2 level cell culture.

Note that full information on the approval of the study protocol must also be provided in the manuscript.

Flow Cytometry

Plots

Confirm that:

☒ The axis labels state the marker and fluorochrome used (e.g. CD4-FITC).

☒ The axis scales are clearly visible. Include numbers along axes only for bottom left plot of group (a 'group' is an analysis of identical markers).

☒ All plots are contour plots with outliers or pseudocolor plots.

☒ A numerical value for number of cells or percentage (with statistics) is provided.

Methodology

Sample preparation: Plasma samples were collected from chick embryos longitudinally, prior to and after intravenous injection of purified extracellular vesicles carrying fluorescent pHluo_M153R-CD63

Instrument: The CellStream from Luminex

Software	Data is acquired with CellStream Acquisition software and analyzed in Cytobank.
Cell population abundance	This analysis involves the quantitative assessment of extracellular vesicles in plasma. There is no purification or sorting applicable in this study.
Gating strategy	Since detection of the extracellular vesicles within the plasma is based on fluorescent detection of injected fluorescently-labeled vesicles, gating is based on the comparison of positive controls (purified vesicles), pre-injection plasma, and post-injection plasma. The gate was set to include the positive vesicles and exclude background signal from the pre-injection plasma.

☒ Tick this box to confirm that a figure exemplifying the gating strategy is provided in the Supplementary Information.
